# The impact of loneliness on quality of life in people with Parkinson’s disease: results from the Survey of Health, Ageing and Retirement in Europe

**DOI:** 10.3389/fmed.2023.1183289

**Published:** 2023-06-23

**Authors:** Tino Prell, Aline Schönenberg, Konstantin G. Heimrich

**Affiliations:** ^1^Department of Geriatrics, Halle University Hospital, Halle, Germany; ^2^Department of Neurology, Jena University Hospital, Jena, Germany

**Keywords:** Parkinson’s disease, loneliness, depression, quality of life, SHARE, UCLA Loneliness Scale

## Abstract

**Background:**

Loneliness is a growing issue for public health in an aging society. However, there is a lack of research on loneliness in people with Parkinson’s disease (PwPD).

**Methods:**

We analyzed cross-sectional and longitudinal data from wave 5 (*N* = 559 PwPD) and 6 (*N* = 442 PwPD) from the Survey of Health, Ageing and Retirement in Europe (SHARE). Loneliness was assessed using the three-item version of the Revised UCLA Loneliness Scale. Descriptive statistics, group comparisons, multiple linear regressions, and generalized estimating equation analysis were performed to explore loneliness prevalence, its relationship with other factors, and its impact on Quality of Life (QoL) in PwPD.

**Results:**

Depending on the used cut-off, the prevalence of loneliness in PwPD ranged from 24.1 to 53.8%. These prevalences were higher compared to people without PD. Loneliness was mainly linked to decreased functional abilities, weaker grip strength, more symptoms of depression, and country of residence. Loneliness was also associated with current QoL and predicts future QoL in PwPD, highlighting its impact on well-being.

**Conclusion:**

Addressing loneliness could potentially improve QoL for PwPD, making it a modifiable risk factor that clinicians and policy-makers should consider.

## 1. Introduction

Loneliness is a complex and individual experience in which a person feels socially disconnected, potentially even when in the presence of others (1, 2). While social isolation is an objective state with minimal social interaction, loneliness is a subjective state of feeling unloved and distant from significant others, close friends, and family (3). Lonely individuals have a more negative perception of the world and anticipate unpleasant social encounters, resulting in retention of negative social information (4).

Loneliness is becoming a growing concern for public health in the aging society (1). It has been linked with various chronic conditions, reduced cognitive function, depressive symptoms, poorer quality of life (QoL), functional decline, and premature mortality (3, 5–10). It is important to note that loneliness can be modified and intervened upon before the onset of poor health (11). However, it is still unclear which direction the relationship between loneliness and worse health outcomes primarily follows. Recent research has shown that Parkinson’s disease (PD) involves several social symptoms, such as difficulties producing emotional facial expressions and speech, which can have negative social consequences and greatly impact the patient’s QoL (12). As PD is a chronic condition that progressively worsens physical and mental function, loneliness is also likely to be an issue. While some previous studies emphasize the importance of social aspects in people with PD (PwPD), loneliness has not been extensively studied previously (12–15).

There are studies that have explored loneliness in PD. The first study by Subramanian et al. revealed that PwPD (*N* = 1,527) who reported being lonely experienced a greater symptom severity than those who were not lonely. Moreover, being lonely was associated with lower QoL (15). However, only cross-sectional data were used and loneliness was assessed by a single dichotomous response to the statement “I am lonely.” Another study compared psychosocial factors between PwPD (*N* = 55) and without PD in the nationally representative German Ageing Survey (16). While PwPD reported lower perceived autonomy, they did not report higher levels of loneliness and perceived social isolation compared to people without PD. Again, only cross-sectional data from one country were used and the sample size of PwPD was quite low. As cause and effect cannot be derived from cross-sectional data, subsequent research with longitudinal data was necessary to confirm these results.

We therefore aimed to explore (1) the prevalence of loneliness, (2) its association with other health-related and psychosocial variables, and (3) its impact on QoL in PwPD in a large European dataset.

## 2. Materials and methods

### 2.1. Data source

Data were taken from waves 5 (2013; *N* = 21,370) (17, 18) and 6 (2015; *N* = 10,774) (19) of the Survey of Health, Ageing and Retirement in Europe (SHARE); for technical details about sampling refer to WP_Series_41_2019_Bergmann_et_al.pdf (share-project.org). SHARE is a multidisciplinary, cross-national, and longitudinal research project comprising more than 120,000 individuals aged ≥50 in European countries.^1^ A person was excluded if she or he was incarcerated, hospitalized, or unable to speak the country’s language(s). SHARE is the largest pan-European social science panel study providing internationally comparable longitudinal data about public health and socio-economic living conditions of European individuals. In wave 5, 559 subjects selected that they have PD. In wave 6, 442 selected to have PD. Among the PwPD in wave 6, 227 subjects already had PD in wave 5 and 215 subjects reported to be newly diagnosed with PD between wave 5 and wave 6. We used data from wave 5 and 6, because only in these waves loneliness was assessed.

### 2.2. Variables

Loneliness: Participants completed the three-item version of the Revised UCLA Loneliness Scale (20). The three items companionship, feeling left out, and isolated are answered on a three point Likert scale (“often,” “some of the time,” “hardly ever or never”). The minimum of the resulting score is three (“not lonely”) and the maximum is nine (“very lonely”). Operationalization in SHARE provides the generated Loneliness Scale variable (*loneliness*) as part of the *gv_health module*. The variable is a sum score based on *mh034_*, *mh035_,* and *mh036_* from the *mental health module*. In general, there is no established threshold for the three-item version of the Revised UCLA Loneliness Scale to categorize people into “lonely” and “not lonely.” Therefore, we estimated the prevalence of loneliness by using different thresholds based on the literature (*loneliness* > 3 points, >4 points, or >5 points) (6, 9, 21–23).

Quality of life (QoL): The Control, Autonomy, Self-realization, and Pleasure scale (CASP-12) (24) is one of the most common internationally used measures for QoL. It is composed of the subscales control, autonomy, self-realization, and pleasure. The 12 items are assessed on a four point Likert scale (“often,” “sometimes,” “rarely,” and “never”) and the resulting sum score ranges from 12 to 48 with higher values indicating better QoL. SHARE provides the CASP-12 variable (*casp*) as a generated variable in the *gv_health module*.

Instrumental activities of daily living (IADL): A modified version of IADL was used in SHARE wave 5 with seven activities (*ph049_*) (25): using a map to get around in a strange place, preparing a hot meal, shopping for groceries, making telephone calls, taking medications, doing work around the house or garden, and managing money. The total score ranges from 0 to 7. The higher the index is, the more difficulties with these activities and the lower the mobility of the respondent. SHARE provides *iadl* as generated variable in the *gv_health module*.

Grip strength: We used grip strength as a general biomarker of poor health status (26). Using a dynamometer, maximum grip strength was measured twice for each hand. The grip strength (*maxgrip*) was derived from the *gv_health module*.

Depressive symptoms: The EURO-D scale (27) consists of the following items: depression, pessimism, suicidality, guilt, sleep, interest, irritability, appetite, fatigue, concentration (on reading or entertainment), enjoyment, and tearfulness. It is summed up in the EURO-D variable (*eurod*) as a generated variable in the *gv_health module*. The maximum score is 12 “very depressed” and the minimum score is zero “not depressed.”

Cognitive function: The 10-words delayed recall test (range 0 to 10; *cf016tot* as generated variables in the *gv_health module*) (28, 29) and verbal fluency (name as many animals as possible in 60 s; *cf010_* as generated variable in the *gv_health module*) (30, 31) were used as measures for cognitive function.

As other variables we considered patients’ age (years), sex, and country of residence. Thereby, countries were considered as clusters (linear regression) or separately (generalized estimating equation, GEE), depending on the statistical analyses used by SPSS. We also included the following variables that can be associated with loneliness based on the literature: Eyesight at distance and while reading (5-point Likert scale; higher values indicating poorer vision) (32), hearing (5-point Likert scale; higher values indicating poorer hearing) (33, 34), education (duration of school education in years) (35), body mass index (BMI) (36), number of chronic diseases (0–9) (37), physical inactivity (*phactiv* in the *gv_health module:* How often do you engage in vigorous physical activity, such as sports, heavy housework, or a job that involves physical labor?; “never vigorous nor moderate,” “other”) (35, 38), and pain perception (*ph084_*: Are you troubled with pain?; “yes,” “no”) (38).

Variables were treated as missing and excluded from the analysis in case of missing information (including “Do not know” and “Refusal”). Additional information on the used scales and multi-item indicators is provided by the SHARE project (39).

### 2.3. Statistical analyses

All analyses were conducted using SPSS (IBM SPSS Statistics, RRID:SCR_016479, version 25) and JASP (JASP, RRID:SCR_015823, version 0.16). The statistical significance was determined with *p* < 0.05. Descriptive statistics were used to characterize the sample. Normality was tested with the Shapiro–Wilk test. Group comparisons between PwPD with low (*loneliness* ≤ 4 points) and high loneliness values (*loneliness* > 4) were performed using *t*-test, *U*-test or *ANOVA* where appropriate. Multiple linear regressions were used to analyze predictors of *loneliness* and QoL (CASP-12) using a stepwise selection algorithm and the Akaike information criterion (AIC) as selection criterion. As independent variables we included the categorical variables sex, country, pain perception, and physical inactivity, and correlating variables with *r* > 0.2 related to *loneliness* or QoL. As two cognitive parameters were recorded (Recall test; Verbal fluency), we considered the one that showed the higher relevant correlation. Multicollinearity was tested using the variance inflation criterion. For longitudinal comparison of all variables that can change over time the Wilcoxon signed-rank and McNemar test were used. The longitudinal association between *loneliness* and QoL was determined using generalized estimating equation (GEE) analysis. GEE enables correction for dependency of observations within individuals over time, by choosing a ‘working’ correlation structure. In the analyses of all models the unstructured working correlation structure was selected as it provided the lowest Quasi-likelihood information criterion (QIC) value. In the adjusted analysis, the baseline predictors associated with QoL in the linear regression were added to the model.

## 3. Results

### 3.1. Prevalence of loneliness

**Figure 1:**
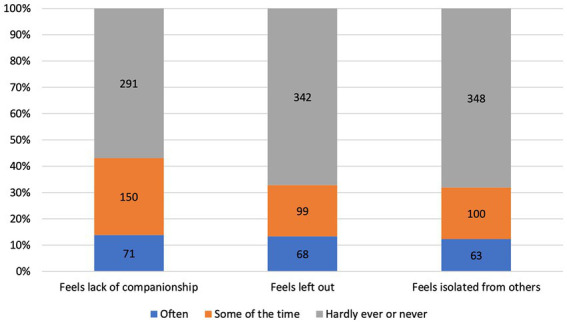
Distribution of loneliness items of PwPD in wave 5. Values indicate the number of people with PD (PwPD) in each subgroup from the entire cohort of PwPD (N = 559). According to the three-item version of the Revised UCLA Loneliness Scale, the items companionship, left out, and isolated are answered on a three point Likert scale (“often,” “some of the time,” “hardly ever or never”).

Descriptive statistics of the 559 PwPD in wave 5 are given in Table 1. The analysis for loneliness on item-level is given in Figure 1, and regarding the sum score in Supplementary Table 1. Using a threshold of 4 points in the Revised UCLA Loneliness Scale (corresponding to the median of *loneliness* = 4 points), we determined a prevalence of loneliness of 37.1% in PwPD and 20.3% in people without PD (see Supplementary Table 1). Based on alternative thresholds, the prevalence of loneliness in PwPD ranged from 24.1% (*loneliness* > 3 points) to 53.8% (*loneliness* > 5 points; see Supplementary Table 1). These prevalences were higher compared to people without PD (11.3% for *loneliness* > 3 points; 37.3% for *loneliness* > 5 points; Supplementary Table 1).

**Table 1:** Descriptive statistics of PwPD in wave 5.

		*N*	%
Sex	Male	291	52.1
	Female	268	47.9
Pain	Yes	357	64.0
	No	201	36.0
Physical inactivity	Other	328	58.7
	Never vigorous nor moderate physical activity	231	41.3
		M	SD
Age at survey (years)		75.67	9.34
Education (years)		10.18	4.60
BMI (kilogram/meter^2^)		26.50	4.76
QoL (score 12–48)		32.93	6.78
Verbal fluency (score)		15.62	8.26
Recall test (score 0–10)		2.35	2.14
Chronic diseases (score 0–9)		3.46	2.05
Depressive symptoms (score 0–12)		4.17	2.68
IADL (score 0–7)		2.28	2.48
Loneliness (score 3–9)		4.45	1.81
Grip strength (kilogram)		28.73	11.42
Eyesight distance (Likert scale 1–5)		3.15	1.15
Eyesight reading (Likert scale 1–5)		3.25	1.15
Hearing (Likert scale 1–5)		3.17	1.06

Values are presented as mean (M), standard deviation (SD); categorical parameters are presented as numbers (*N*) and percentages (%). Age of people with Parkinson’s disease (PwPD) is presented in years. BMI: Body Mass Index; Chronic diseases: Number of chronic diseases (ranging from 0 to 9); Depressive symptoms: according to the EURO-D scale (ranging from 0 to 12, higher values indicate more depressive symptoms); Education: duration of school education in years; Eyesight distance: 5-point Likert scale, higher values indicating poorer vision at distance; Eyesight reading: 5-point Likert scale, higher values indicating poorer vision when reading; Grip strength: maximum hand grip strength given in kilogram; Hearing: 5-point Likert scale, higher values indicating poorer hearing; IADL: Instrumental Activities of Daily Living (ranging from 0 to 7, higher values indicate impaired mobility); Loneliness: Sum score of the three-item version of the Revised UCLA Loneliness Scale (ranging from 3 to 9, higher values indicate more loneliness); Pain: dichotomous variable (Are you troubled with pain?); Physical inactivity: dichotomous variable (How often do you engage in vigorous physical activity, such as sports, heavy housework, or a job that involves physical labor?); Recall test: 10-words delayed recall test (ranging from 0 to 10, higher values indicate better cognitive functioning); QoL: Quality of Life according to the Control, Autonomy, Self-realization, and Pleasure (CASP-12) scale (ranging from 12 to 48, higher values indicate better quality of life); Verbal fluency: number of named animals.

### 3.2. Factors associated with loneliness

In the univariate analyses a higher loneliness score was associated with higher age, more *chronic diseases*, poorer cognitive function, more *depressive symptoms*, more limitations in *IADL*, lower *grip strength*, more *physical inactivity*, higher frequency of *pain*, more sensory problems (*hearing*, *vision*), and lower *QoL* in PwPD (Table 2); with strongest correlation for *depressive symptoms* and *QoL*. The categorical variables *sex*, *country*, *pain* perception, *physical inactivity* and the correlating variables with r > 0.2 (see Supplementary Table 2) were entered into a stepwise linear regression with *loneliness* as dependent variable. Here, *depressive symptoms*, *country*, *IADL*, and *grip strength* explained 32% of variance of *loneliness* [*F*(6, 500) = 40.55, *p* < 0.001; Table 3].

**Table 2:** Comparison of PwPD with low or high loneliness values in wave 5.

		*Loneliness* ≤ 4		*Loneliness* > 4		Sig	Effect size
		*N*	%	*N*	%	*p*	Phi
Sex	Male	176a	55.2	87a	46.3	0.053	0.086
	Female	143a	44.8	101a	53.7		
Pain	Yes	182a	57.1	140b	74.5	<0.001	−0.175
	No	137a	42.9	48b	25.5		
Physical inactivity	Other	227a	71.2	92b	48.9	<0.001	0.222
	Never vigorous nor moderate physical activity	92a	28.8	96b	51.1		
		M	SD	M	SD	*p*	Cohen’s d
Age at survey (years)		74.36a	8.97	76.35b	9.35	0.018	−0.218
Education (years)		10.74a	4.75	9.28a	4.24	0.056	0.321
BMI (kilogram/meter^2^)		26.39a	4.48	27.00a	4.89	0.170	−0.130
QoL (score 12–48)		35.28a	6.10	28.75b	5.95	<0.001	1.080
Verbal fluency (score)		17.15a	7.84	13.49b	8.19	<0.001	0.460
Recall test (score 0–10)		2.63a	2.15	1.98b	2.05	0.001	0.306
Chronic diseases (score 0–9)		3.09a	1.94	3.87b	2.10	<0.001	0.390
Depressive symptoms (score 0–12)		3.33a	2.37	5.63b	2.55	<0.001	−0.942
IADL (score 0–7)		1.38a	1.96	2.99b	2.41	<0.001	−0.752
Grip strength (kilogram)		30.21a	11.61	25.75b	10.41	<0.001	0.397
Eyesight distance (Likert scale 1–5)		2.88a	1.08	3.40b	1.14	<0.001	−0.469
Eyesight reading (Likert scale 1–5)		3.02a	1.12	3.42b	1.12	<0.001	−0.361
Hearing (Likert scale 1–5)		3.01a	1.02	3.27b	1.11	0.004	−0.248

Values are presented as mean (M), standard deviation (SD); categorical parameters are presented as numbers (*N*) and percentages (%). Age of people with Parkinson’s disease (PwPD) is presented in years. BMI: Body Mass Index; Chronic diseases: Number of chronic diseases (ranging from 0 to 9); Depressive symptoms: according to the EURO-D scale (ranging from 0 to 12, higher values indicate more depressive symptoms); Education: duration of school education in years; Eyesight distance: 5-point Likert scale, higher values indicating poorer vision at distance; Eyesight reading: 5-point Likert scale, higher values indicating poorer vision when reading; Grip strength: maximum hand grip strength given in kilogram; Hearing: 5-point Likert scale, higher values indicating poorer hearing; IADL: Instrumental Activities of Daily Living (ranging from 0 to 7, higher values indicate impaired mobility); Loneliness: Sum score of the three-item version of the Revised UCLA Loneliness Scale (ranging from 3 to 9, higher values indicate more loneliness); Pain: dichotomous variable (Are you troubled with pain?); Physical inactivity: dichotomous variable (How often do you engage in vigorous physical activity, such as sports, heavy housework, or a job that involves physical labor?); Recall test: 10-words delayed recall test (ranging from 0 to 10, higher values indicate better cognitive functioning); QoL: Quality of Life according to the Control, Autonomy, Self-realization, and Pleasure (CASP-12) scale (ranging from 12 to 48, higher values indicate better quality of life); Verbal fluency: number of named animals. Note: Values in the same row and sub-table where the subscript is not identical differ strongly at *p* < 0.05 in the two-tailed test for equality for column means. Effect sizes are presented as Phi (low: 0.1; moderate: 0.3; strong: 0.5) for categorical variables and Cohen’s d (low: 0.2; moderate: 0.5; strong: 0.8) for metrical variables.

**Table 3:** Predictors of loneliness in the linear regression in wave 5.

	Coefficient	*p*	Beta
Constant	3.52	<0.001	
Depressive symptoms	0.206	<0.001	0.393
Country (Italy)	1.435	<0.001	0.390
Country (Germany, Israel, Czech Republic)	0.689	<0.001	0.390
Country (Austria, Netherlands, Denmark, Switzerland, Slovenia)	−0.375	0.032	0.390
Country (Sweden, Spain, France, Belgium, Luxembourg, Estonia)	0*	-	0.390
IADL	0.165	<0.001	0.185
Grip strength	−0.015	0.041	0.032

Stepwise selection with Akaike information criterion (AIC). Dependent variable: Loneliness. Entered independent variables: Sex, Country, Pain, Physical inactivity, Verbal fluency, Depressive symptoms, IADL, Grip strength, Eyesight distance. Values are presented as standardized beta coefficients. Depressive symptoms: according to the EURO-D scale (ranging from 0 to 12, higher values indicate more depressive symptoms); Eyesight distance: higher values indicating poorer vision at distance; Grip strength: maximum hand grip strength given in kilogram; IADL: Instrumental Activities of Daily Living (ranging from 0 to 7, higher values indicate impaired mobility); Loneliness: Sum score of the three-item version of the Revised UCLA Loneliness Scale (ranging from 3 to 9, higher values indicate more loneliness); Pain: dichotomous variable (Are you troubled with pain?); Physical inactivity: dichotomous variable (How often do you engage in vigorous physical activity, such as sports, heavy housework, or a job that involves physical labor?); Verbal fluency: number of named animals.

* Reference point set to zero.

### 3.3. Association between loneliness and QoL: cross-sectional

We then examined the impact of *loneliness* on *QoL*. *Loneliness* explained 24% of the *QoL* variance [*F*(1, 468) = 148.18, *p* < 0.001] and remained an important predictor of *QoL* after adjustment for cofactors [beta = 0.09; *p* < 0.001; *F*(10, 459) = 66.35, *p* < 0.001; Supplementary Table 3].

### 3.4. Predictors of QoL: longitudinal

Regarding the temporal dynamics, the following parameters changed between wave 5 and wave 6 in PwPD who received assessments at both waves (*N* = 227; 55.9% male; mean age 74.3 ± 8.4 years at wave 5): *Loneliness* increased, more people felt lonely (number of people with *loneliness* > 4), *BMI* decreased, *QoL* worsened, *verbal fluency* worsened, and *depressive symptoms* increased. Moreover, PwPD reported more limitations in *IADL*, more *physical inactivity*, and had a lower *grip strength* (Supplementary Table 4). Further analysis was conducted to study the longitudinal association between *loneliness* and *QoL* using GEE analysis adjusted for the factors that have been associated with *QoL* in the linear regression (i.e., *depressive symptoms*, *IADL*, *verbal fluency*, *hearing*, *eyesight distance*, *country*). In this GEE model, *loneliness*, *depressive symptoms*, *IADL*, *verbal fluency*, and *country* made significant independent contributions to *QoL* (Table 4). Few variables such as *eyesight distance* and *hearing* were not statistically significant in the adjusted model.

**Table 4:** Predictors of future QoL (CASP-12), GEE analysis.

Parameter	B	Std. error	95% Wald confidence interval		Hypothesis test			Exp(B)	95% Wald confidence interval for Exp(B)	
			Lower	Upper	Wald Chi-square	df	Sig.		Lower	Upper
(Intercept)	38.278	1.6036	35.135	41.421	569.740	1	0.000	42054665843378912.000	1814638086940400.800	974626804058889980.000
Depressive symptoms	−0.809	0.1022	−1.010	−0.609	62.634	1	<0.001	0.445	0.364	0.544
IADL	−0.666	0.1212	−0.904	−0.429	30.236	1	<0.001	0.514	0.405	0.651
Loneliness	−0.738	0.1778	−1.087	−0.390	17.250	1	<0.001	0.478	0.337	0.677
Eyesight distance	−0.140	0.2358	−0.602	0.322	0.354	1	0.552	0.869	0.547	1.380
Hearing	−0.306	0.2330	−0.762	0.151	1.722	1	0.189	0.737	0.466	1.163
Verbal fluency	0.101	0.0438	0.016	0.187	5.356	1	0.021	1.107	1.016	1.206
Austria	3.044	0.9920	1.100	4.989	9.416	1	0.002	20.993	3.004	146.729
Germany	4.224	1.2457	1.782	6.665	11.495	1	<0.001	68.279	5.942	784.621
Sweden	4.812	0.9367	2.976	6.648	26.396	1	<0.001	123.018	19.619	771.370
Spain	1.569	0.9013	−0.197	3.336	3.032	1	0.082	4.803	0.821	28.103
Italy	−0.103	1.3571	−2.763	2.557	0.006	1	0.940	0.902	0.063	12.898
France	2.967	1.0493	0.910	5.023	7.993	1	0.005	19.427	2.484	151.903
Denmark	4.841	1.2061	2.477	7.205	16.112	1	<0.001	126.632	11.910	1346.391
Switzerland	3.946	1.2549	1.486	6.406	9.887	1	0.002	51.727	4.421	605.248
Belgium	2.460	1.0722	0.358	4.561	5.262	1	0.022	11.700	1.431	95.688
Israel	1.316	1.2309	−1.096	3.729	1.144	1	0.285	3.730	0.334	41.634
Czech Republic	0.370	0.9060	−1.406	2.146	0.167	1	0.683	1.447	0.245	8.547
Luxembourg	7.040	1.6444	3.817	10.263	18.326	1	<0.001	1140.952	45.448	28643.246
Slovenia	3.138	1.1416	0.900	5.375	7.554	1	0.006	23.049	2.460	215.959
Estonia	0^a^	.	.	.	.	.	.	1	.	.
(Scale)	17.820									

Parameter estimates of the generalized estimating equation (GEE; Goodness of fit: QIC = 6265.851). Dependent variable: QoL (CASP-12) in wave 6. Independent variables from wave 5. Model: (Intercept), Depressive symptoms, IADL, Loneliness, Eyesight distance, Hearing, Verbal fluency, Country (Reference: Estonia). Depressive symptoms: according to the EURO-D scale (ranging from 0 to 12, higher values indicate more depressive symptoms); Eyesight distance: 5-point Likert scale, higher values indicating poorer vision at distance; Hearing: 5-point Likert scale, higher values indicating poorer hearing; IADL: Instrumental Activities of Daily Living (ranging from 0 to 7, higher values indicate impaired mobility); Loneliness: Sum score of the three-item version of the Revised UCLA Loneliness Scale (ranging from 3 to 9, higher values indicate more loneliness); QoL: Quality of Life according to the Control, Autonomy, Self-realization, and Pleasure (CASP-12) scale (ranging from 12 to 48, higher values indicate better quality of life); Verbal fluency: number of named animals. a: Set to 0, as Estonia is used as reference.

## 4. Discussion

With this study, we aimed to determine the (1) prevalence of loneliness, (2) its association with other variables, and (3) its impact on QoL in PwPD in European countries.

The study showed that loneliness was prevalent among PwPD, with a range of 24.1 to 53.8% affected depending on the threshold used to define loneliness. It is important to note that there is no established threshold for the Revised UCLA Loneliness Scale, but in this study, loneliness was defined as endorsing at least “some of the time” to two items or at least “often” to one of the three loneliness items. This led to a prevalence of 37.1% (*loneliness* > 4). Regardless of the definition used, PwPD were consistently more likely to be classified as lonely than those without PD in the study.

Perissinotto et al. conducted an international study to determine the prevalence of loneliness among people aged 60 or above. They used a definition of loneliness that classified respondents as lonely if their loneliness score was above 3 (corresponding threshold of the Revised UCLA Loneliness Scale: *loneliness* > 3). The results showed that 43.2% of the respondents of the Health and Retirement Study (HRS) in the United States reported feeling lonely (9). Gerst-Emerson and Jayawardhana reported even higher rates of loneliness (corresponding threshold of the Revised UCLA Loneliness Scale: *loneliness* > 3), with 52.7 and 56.6% of HRS respondents reporting loneliness in 2008 and 2012, respectively (21). A comparison with European studies showed that 45.1% of the English Longitudinal Study of Ageing (ELSA) wave 5 respondents felt lonely (corresponding threshold of the Revised UCLA Loneliness Scale: *loneliness* > 3) (23). Using another definition of loneliness (corresponding threshold of the Revised UCLA Loneliness Scale: *loneliness* > 5) different studies found that between 17.0 and 18.1% of respondents reported feeling lonely (6, 22, 23).

It should be noted that the prevalence of loneliness varies depending on the measure of loneliness used, the population studied, the age group considered, and the sample size. These factors may also affect the severity of loneliness observed (3). However, using the three-item version of the Revised UCLA Loneliness Scale our study found that PwPD had a mean sum score of 4.45 ± 1.81, which is higher than the scores reported from ELSA (mean sum scores of less than 4.12) (6, 22, 40). These results indicate that loneliness is a common and more severe health issue in PwPD compared to the general population.

Research conducted previously has shown that loneliness can have significant negative effects on the physical and mental health of older adults, and can even lead to an increased risk of mortality. In PwPD, one cross-sectional study revealed that being lonely was associated with lower QoL (15). Thereby, loneliness was assessed by a single item. However, due to the variety of the clinical picture, loneliness should rather be seen as a continuous and multidimensional symptom. In this regard, the use of a scale, e.g., the Revised UCLA Loneliness Scale, is more preferable. Our findings from cross-sectional data from wave 5 suggests that loneliness is strongly linked with a range of functional impairments, including lower grip strength and more depressive symptoms. Additionally, our findings indicate that the country in which a person lives can also play a significant role in their experience of loneliness and associated health outcomes. This is consistent with previous research showing that feelings of loneliness among older people are more common in southern Europe than in its northern parts (41–43). Accordingly, loneliness is more common in areas where community ties are considered stronger, which suggests that loneliness is related to a change in community ties rather than the level of social ties *per se* (44). Moreover, feeling lonely may depend on different access to healthcare provision, psychotherapy or psychosocial support, and cultural barriers to healthcare, and may therefore vary among countries. However, in addition to cultural factors and population characteristics, individual factors are also contributing to loneliness. Thereby, previous studies revealed that in particular living alone and having poor health are important risk factors for loneliness (41, 42). In addition to loneliness as a measure of social exclusion, differences among countries have already been demonstrated for deprivation (45, 46). Nevertheless, this was the first study that showed differences in loneliness among PwPD between different countries. As these country-specific variations cannot be interpreted as causal effects, further research is needed to examine these differences in larger samples and to identify country-specific reasons for a lesser or greater impact of loneliness on physical or mental health in PwPD.

The SHARE dataset includes a broad range of older adults and does not focus on any particular conditions. As a result, there are no assessments specifically tailored for measuring motor functions related to PD. Instead, our study utilized IADL and grip strength as substitutes for functioning since difficulties in everyday tasks are often linked to PD. (47–49) Lonely individuals are more prone to experiencing declines in daily activities (9) and grip strength can be utilized as a general measure of poor health status (26), which is also associated with PD severity (50, 51).

The outcomes of our study emphasize the connection between feelings of loneliness and symptoms of depression in individuals with PD. In the general population, loneliness is seen as a risk factor of depression (52), especially in old age (6). Research conducted on the ELSA group revealed that eliminating loneliness could prevent almost 18% of depression cases (6). However, there is evidence of a bidirectional relationship between loneliness and depression in older adults (53–55). As loneliness is of major importance in PwPD, one can assume that a two-way relationship can be suspected between loneliness and depression in PwPD as well. Nonetheless, to determine whether loneliness plays a causal role in the development of depression in PwPD, a more extensive longitudinal study is required.

Our research has identified that loneliness has a negative impact on the QoL of PwPD, both in the present and in particular the future, affecting both physical and mental health. The study also highlights that depressive symptoms are known to be determinants of low QoL in PwPD (56–58), but the longitudinal data reveals that loneliness has an equally detrimental effect on QoL. Additionally, we found that loneliness and depressive symptoms contribute independently to QoL. The study underscores the clinical significance of loneliness in PwPD, as difficulties in producing emotional facial expressions or speech can lead to severe negative social consequences, further affecting QoL (12, 59).

Loneliness is a risk factor for the well-being of PwPD, and as such, it should be considered when making treatment decisions. Finding ways to reduce or prevent loneliness in these individuals can lead to an improvement in their QoL. However, there is no uniform way of evaluating and documenting loneliness as a social determinant of health in older adults (60). Therefore, it is crucial to raise awareness and accurate screen for loneliness among PwPD (61), which can be done for example by either specific screening questions (15) or regularly using the Revised UCLA Loneliness Scale. The next step in addressing loneliness among PwPD is to improve existing knowledge about the interventions that have been shown to be reasonably effective in older adults (61). In this regard it is important to determine whether direct interventions such as changing thought patterns (e.g., Cognitive Behavioral Therapy) (62), social skills training and psychoeducation, supportive socialization to select and attend activities, and wider community groups to create a connectedness in the community itself (e.g., social prescribing) (63, 64) or indirect approaches such as treating depression, are more advantageous (65). Considering the fact that loneliness is a subjective feeling, a holistic treatment approach must take into account the patient’s needs and acceptance in addition to causal considerations.

In our research, there are certain limitations that need to be acknowledged. First, the diagnosis of PD is based on self-reporting, which leaves room for the possibility that some individuals who reported PD in wave 6 may have already had the disease or early signs of PD in wave 5. In addition, bias may also exist because the indication of the disease may be preliminary and not validated by a specialist with exclusion of other differential diagnoses. As a result, we must be careful when interpreting longitudinal analyses. Second, widely used measures for PD, such as the Hoehn and Yahr scale, the Movement Disorder Society Unified Parkinson’s Disease Rating Scale, or other nonmotor symptoms are not present in the SHARE dataset. However, we considered everyday functioning and general health. Third, besides the 10-words delayed recall test and the assessment of verbal fluency, no widely used measures of cognitive function (e.g., MMST or MoCA) were used. Accordingly, no distinction could be made between people with or without cognitive impairments based on established thresholds. However, the aforementioned measures are often used in large cohort studies. Moreover, we did not exclude people with other chronic conditions. Accordingly, we cannot exclude that certain diseases may have a potential confounding effect on both QoL and loneliness. Furthermore, our study relies on data from waves 5 and 6 of SHARE, which took place in 2013 and 2015, respectively. Nonetheless, the COVID-19 pandemic has had a significant impact on psychosocial factors. Thus, it would be valuable to investigate loneliness in PwPD using more recent data.

According to our research, loneliness is a prevalent issue affecting the health of PwPD more frequently and severely than the general population. Our findings indicate that loneliness is linked with decreased physical and mental health and has a significant impact on the present and future QoL of PwPD. Therefore, it is essential to address and reduce loneliness as a modifiable risk factor for the well-being of PwPD, and healthcare professionals and policymakers should consider this when making treatment decisions.

## Data availability statement

The data analyzed in this study is subject to the following licenses/restrictions: Data was obtained from the Survey of Health, Ageing and Retirement in Europe (SHARE) and after successful application. Requests to access these datasets should be directed to http://www.share-project.org.

## Ethics statement

The SHARE data collection procedures are subject to continuous ethics review by responsible ethics committees (University of Mannheim and Max Planck Society, Germany), as well as national ethics committees in participating countries.

## Author contributions

TP: study concept, design, statistical analysis, and interpretation of the data. TP and KH: first draft of the manuscript. AS: critical revision of the manuscript. All authors contributed to the article and approved the submitted version.

## Funding

Funding to KH was provided by the Deutsche Forschungsgemeinschaft (DFG, German Research Foundation) in the Clinician Scientist-Program OrganAge, funding number 413668513. Additionally, funding to KH was provided by the Interdisciplinary Center of Clinical Research of the Medical Faculty of Jena. TP received funding from a Bundesministerium für Bildung und Forschung (BMBF) grant (01GY2301).

## Conflict of interest

The authors declare that the research was conducted in the absence of any commercial or financial relationships that could be construed as a potential conflict of interest.

## Publisher’s note

All claims expressed in this article are solely those of the authors and do not necessarily represent those of their affiliated organizations, or those of the publisher, the editors and the reviewers. Any product that may be evaluated in this article, or claim that may be made by its manufacturer, is not guaranteed or endorsed by the publisher.

## Footnotes

^1^www.share-project.org
