# Supplementary material for: The impact of loneliness on quality of life in people with Parkinson’s disease: results from the Survey of Health, Ageing and Retirement in Europe
Source: Front Med (Lausanne). 2023 Jun 23;10:1183289. doi: 10.3389/fmed.2023.1183289 (PMC10326722; doi:10.3389/fmed.2023.1183289)
Supplement: Supplementary file 1 [file Data_Sheet_1.PDF]

Supplementary Material

Supplementary Table 1: Loneliness sum score and prevalence of people with and without PD in wave 5.

<table>
<tr><th colspan="2" rowspan="3">Loneliness sum score</th><th colspan="5">PD</th><th colspan="5">No PD</th></tr>
<tr><th rowspan="2">N</th><th rowspan="2">%</th><th colspan="3">lonely (%)</th><th rowspan="2">N</th><th rowspan="2">%</th><th colspan="3">lonely (%)</th></tr>
<tr><th>A</th><th>B</th><th>C</th><th>A</th><th>B</th><th>C</th></tr>
<tr><td rowspan="8">Valid</td><td>3</td><td>234</td><td>46.2</td><td></td><td></td><td></td><td>40067</td><td>62.7</td><td></td><td></td><td></td></tr>
<tr><td>4</td><td>85</td><td>16.8</td><td rowspan="6">53.8</td><td></td><td></td><td>10857</td><td>17.0</td><td rowspan="6">37.3</td><td></td><td></td></tr>
<tr><td>5</td><td>66</td><td>13.0</td><td rowspan="5">37.1</td><td></td><td>5736</td><td>9.0</td><td rowspan="5">20.3</td><td></td></tr>
<tr><td>6</td><td>45</td><td>8.9</td><td rowspan="4">24.1</td><td>3752</td><td>5.9</td><td rowspan="4">11.3</td></tr>
<tr><td>7</td><td>27</td><td>5.3</td><td>1702</td><td>2.7</td></tr>
<tr><td>8</td><td>24</td><td>4.7</td><td>750</td><td>1.2</td></tr>
<tr><td>9</td><td>26</td><td>5.1</td><td>1019</td><td>1.6</td></tr>
<tr><td>Total</td><td>507</td><td>100.0</td><td></td><td></td><td></td><td>63883</td><td>100.0</td><td></td><td></td><td></td></tr>
<tr><td>Missing</td><td>System</td><td>52</td><td colspan="4" rowspan="2"></td><td>1480</td><td colspan="4" rowspan="2"></td></tr>
<tr><td colspan="2">Total</td><td>559</td><td>65363</td></tr>
</table>

Values indicate the distribution of the loneliness sum score according to the three-item version of the revised UCLA loneliness scale (ranging from 3 to 9, higher values indicate more loneliness), and are presented as numbers (N) and percentages (%) of people with or without Parkinson's disease (PD). In addition, the prevalences of loneliness are reported based on three different thresholds of loneliness; accordingly, people are classified as lonely, if they received a loneliness sum score of more than three (A), four (B), or five points (C).

Supplementary Table 2: Spearman's rho correlation matrix of people with PD in wave 5.

	Loneliness	Age	Education	BMI	QoL	Verbal fluency	Recall test	Chronic diseases	Depressive symptoms	IADL	Grip strength	Eyesight distance	Eyesight reading	Hearing
Loneliness	1	,155**	-0,134	0,036	-,483**	-,240**	-,178**	,193**	,428**	,392**	-,218**	,241**	,192**	,127**
Age	,155**	1	-,263**	-,162**	-,226**	-,399**	-,394**	,212**	,115*	,388**	-,349**	,331**	,307**	,321**
Education	-0,134	-,263**	1	-0,154	,371**	,399**	,291**	-,161*	-,343**	-,311**	,279**	-,365**	-,438**	-,326**
BMI	0,036	-,162**	-0,154	1	-0,058	,097*	0,07	,149**	0,021	-,109*	0,077	-0,051	-0,018	0,052
QoL	-,483**	-,226**	,371**	-0,058	1	,435**	,323**	-,212**	-,600**	-,503**	,296**	-,392**	-,325**	-,266**
Verbal fluency	-,240**	-,399**	,399**	,097*	,435**	1	,568**	-,177**	-,325**	-,503**	,387**	-,320**	-,314**	-,248**
Recall test	-,178**	-,394**	,291**	0,07	,323**	,568**	1	-,148**	-,250**	-,408**	,283**	-,273**	-,276**	-,178**
Chronic diseases	,193**	,212**	-,161*	,149**	-,212**	-,177**	-,148**	1	,227**	,299**	-,275**	,252**	,219**	,162**
Depressive symptoms	,428**	,115*	-,343**	0,021	-,600**	-,325**	-,250**	,227**	1	,452**	-,241**	,322**	,262**	,166**
IADL	,392**	,388**	-,311**	-,109*	-,503**	-,503**	-,408**	,299**	,452**	1	-,376**	,412**	,387**	,223**
Grip strength	-,218**	-,349**	,279**	0,077	,296**	,387**	,283**	-,275**	-,241**	-,376**	1	-,254**	-,169**	-0,095
Eyesight distance	,241**	,331**	-,365**	-0,051	-,392**	-,320**	-,273**	,252**	,322**	,412**	-,254**	1	,672**	,374**
Eyesight reading	,192**	,307**	-,438**	-0,018	-,325**	-,314**	-,276**	,219**	,262**	,387**	-,169**	,672**	1	,376**
Hearing	,127**	,321**	-,326**	0,052	-,266**	-,248**	-,178**	,162**	,166**	,223**	-0,095	,374**	,376**	1

Correlation matrix of variables using Spearman's correlation coefficient. Significant correlations are indicated by *(*$p < 0.05$, **$p < 0.01$). Age: participants age at survey in years; BMI: Body Mass Index; Chronic diseases: Number of chronic diseases (ranging from 0 to 9); Depressive symptoms: according to the EURO-D scale (ranging from 0 to 12, higher values indicate more depressive symptoms); Education: duration of school education in years; Eyesight distance: 5-point Likert scale, higher values indicating poorer vision at distance; Eyesight reading: 5-point Likert scale, higher values indicating poorer vision when reading; Grip strength: maximum hand grip strength given in kilogram; Hearing: 5-point Likert scale, higher values indicating poorer hearing; IADL: Instrumental Activities of Daily Living (ranging from 0 to 7, higher values indicate impaired mobility); Loneliness: Sum score of the three-item version of the Revised UCLA Loneliness Scale (ranging from 3 to 9, higher values indicate more loneliness); Recall test:10-words delayed recall test (ranging from 0 to 10, higher values indicate better cognitive functioning); QoL: Quality of life according to the Control, Autonomy, Self-realization, and Pleasure scale (CASP-12) (ranging from 12 to 48, higher values indicate better quality of life); Verbal fluency: number of named animals.

Supplementary Table 3: Predictors of quality of life (CASP-12) in the linear regression in wave 5.

	Coefficient	P	beta
Constant	44,237	<.001	
EURO-D	-0,939	<.001	0.458
Country (Italy)	-4,500	<.001	0.259
Country (Spain, France, Estonia)	-3,272	<.001	0.259
Country (Austria, Germany, Belgium, Slovenia)	-1,664	.007	0.259
Country (Israel, Czech Republic)	-5,087	<.001	0.259
Country (Sweden, Netherlands, Denmark, Switzerland, Luxembourg)	0*	-	0.259
IADL	-0,570	<.001	0.112
Loneliness	-0,591	<.001	0.086
Verbal fluency	0,098	0.002	0.043
Hearing	-0,533	0.011	0.029
Eyesight distance	-0,366	0.083	0.013

Stepwise selection with Akaike information criterion (AIC). Dependent variable: QoL. Entered independent variables: Sex, Country, Pain, Physical inactivity, Age, Education, Verbal fluency, Chronic diseases, Depressive symptoms, IADL, Loneliness, Grip strength, Eyesight distance, Eyesight reading, Hearing. Values are presented as standardized beta coefficients. Age: Participants' age at survey; Chronic diseases: Number of chronic diseases (ranging from 0 to 9); Depressive symptoms: according to the EURO-D scale (ranging from 0 to 12, higher values indicate more depressive symptoms); Education: duration of school education in years; Eyesight distance: 5-point Likert scale, higher values indicating poorer vision at distance; Eyesight reading: 5-point Likert scale, higher values indicating poorer vision when reading; Grip strength: maximum hand grip strength given in kilogram; Hearing: 5-point Likert scale, higher values indicating poorer hearing; IADL: Instrumental Activities of Daily Living (ranging from 0 to 7, higher values indicate impaired mobility); Loneliness: Sum score of the three-item version of the Revised UCLA Loneliness Scale (ranging from 3 to 9, higher values indicate more loneliness); Pain: dichotomous variable (Are you troubled with pain?); Physical inactivity: dichotomous variable (How often do you engage in vigorous physical activity, such as sports, heavy housework, or a job that involves physical labour?); QoL: Quality of Life according to the Control, Autonomy, Self-realization, and Pleasure (CASP-12) scale (ranging from 12 to 48, higher values indicate better quality of life); Verbal fluency: number of named animals. *reference point set to zero.

Supplementary Table 4: Comparison of PwPD between wave 5 and wave 6.

		Wave 5		Wave 6				
		N	%	N	%	Mc Nemar test		
						P	X^2	Log OR
Loneliness	≤ 4	147	67.7	116	59.2	0.011	6.48	0.75
	> 4	70	32.3	80	40.8			
Pain	Yes	139	61.2	147	64.8	0.248	1.33	-
	No	88	38.8	80	35.2			
Physical inactivity	Other	159	70.0	139	61.2	0.008	7.14	0.74
	Never vigorous nor moderate physical activity	68	30.0	88	38.8			
		M	SD	M	SD	Wilcoxon signed rank test		
						P	Z	r
BMI (kilogram/meter^2)		26.57	5.03	26.39	5.40	0.004	-2.883	0.191
QoL (score 12-48)		33.71	6.56	33.04	6.19	0.007	-2.684	0.178
Verbal fluency (score)		17.29	7.61	16.74	6.93	0.003	-2.931	0.195
Recall test (score 0-10)		2.68	2.22	2.80	1.94	0.342		
Chronic diseases (score 0-9)		3.19	1.95	3.44	2.01	0.082		
Depressive symptoms (score 0-12)		3.78	2.39	4.00	2.43	0.025	-2.239	0.149
IADL (score 0-7)		1.63	2.08	3.27	3.24	<.001	-8.932	0.593
Loneliness (score 3-9)		4.22	1.63	4.44	1.63	0.010	-2.579	0.171
Grip strength (kilogram)		29.66	11.79	28.69	11.19	<.001	-4.284	0.284
Eyesight distance (Likert scale 1-5)		3.02	1.11	3.04	1.04	0.954		
Eyesight reading (Likert scale 1-5)		3.12	1.13	3.16	1.02	0.618		
Hearing (Likert scale 1-5)		3.10	1.07	3.14	1.11	0.442		

Values are presented as mean (M), standard deviation (SD); categorical parameters are presented as numbers (N) and percentages (%). BMI: Body Mass Index; Chronic diseases: Number of chronic diseases (ranging from 0 to 9); Depressive symptoms: according to the EURO-D scale (ranging from 0 to 12, higher values indicate more depressive symptoms); Eyesight distance: 5-point Likert scale, higher values indicating poorer vision at distance; Eyesight reading: 5-point Likert scale, higher values indicating poorer vision when reading; Grip strength: maximum hand grip strength given in kilogram; Hearing: 5-point Likert scale, higher values indicating poorer hearing; IADL: Instrumental Activities of Daily Living (ranging from 0 to 7, higher values indicate impaired mobility); Loneliness: Sum score of the three-item version of the Revised UCLA Loneliness Scale (ranging from 3 to 9, higher values indicate more loneliness); Pain: dichotomous variable (Are you troubled with pain?); Physical inactivity: dichotomous variable (How often do you engage in vigorous physical activity, such as sports, heavy housework, or a job that involves physical labour?); Recall test: 10-words delayed recall test (ranging from 0 to 10, higher values indicate better cognitive functioning); QoL: Quality of Life according to the Control, Autonomy, Self-realization, and Pleasure (CASP-12) scale (ranging from 12 to 48, higher values indicate better quality of life); Verbal fluency: number of named animals. Effect sizes are calculated via $r = Z/\sqrt{N}$ (low: 0.1; moderate: 0.3; strong: 0.5) and are based on N = 227 people with Parkinson's disease (PwPD) who received assessments at both waves.
